# Development of a Management App for Postviral Fibromyalgia-Like Symptoms: Patient Preference-Guided Approach

**DOI:** 10.2196/50832

**Published:** 2024-04-19

**Authors:** Marc Blanchard, Cinja Nadana Koller, Pedro Ming Azevedo, Tiffany Prétat, Thomas Hügle

**Affiliations:** 1 Department of Rheumatology Lausanne University Hospital (CHUV) University of Lausanne Lausanne Switzerland

**Keywords:** digital health, patient preference, user experience, patient-centricity, platform, development, fibromyalgia, self-management, quality of life, patient outcome, musculoskeletal, usability testing, digital health solution

## Abstract

**Background:**

Persistent fibromyalgia-like symptoms have been increasingly reported following viral infections, including SARS-CoV-2. About 30% of patients with post–COVID-19 syndrome fulfill the fibromyalgia criteria. This complex condition presents significant challenges in terms of self-management. Digital health interventions offer a viable means to assist patients in managing their health conditions. However, the challenge of ensuring their widespread adoption and adherence persists. This study responds to this need by developing a patient-centered digital health management app, incorporating patient preferences to enhance usability and effectiveness, ultimately aiming to improve patient outcomes and quality of life.

**Objective:**

This research aims to develop a digital health self-management app specifically for patients experiencing postviral fibromyalgia-like symptoms. By prioritizing patient preferences and engagement through the app’s design and functionality, the study intends to facilitate better self-management practices and improve adherence.

**Methods:**

Using an exploratory study design, the research used patient preference surveys and usability testing as primary tools to inform the development process of the digital health solution. We gathered and analyzed patients’ expectations regarding design features, content, and usability to steer the iterative app development.

**Results:**

The study uncovered crucial insights from patient surveys and usability testing, which influenced the app’s design and functionality. Key findings included a preference for a symptom list over an automated chatbot, a desire to report on a moderate range of symptoms and activities, and the importance of an intuitive onboarding process. While usability testing identified some challenges in the onboarding process, it also confirmed the importance of aligning the app with patient needs to enhance engagement and satisfaction.

**Conclusions:**

Incorporating patient feedback has been a significant factor in the development of the digital health app. Challenges encountered with user onboarding during usability testing have highlighted the importance of this process for user adoption. The study acknowledges the role of patient input in developing digital health technologies and suggests further research to improve onboarding procedures, aiming to enhance patient engagement and their ability to manage digital health resources effectively.

**International Registered Report Identifier (IRRID):**

RR2-10.2196/32193

## Introduction

### Postviral Syndromes, Post–COVID-19, and Fibromyalgia-Like Symptoms

Postviral syndromes have been substantially observed and described in the past. Epstein-Barr virus or Q fever has for example led patients to develop chronic symptoms such as fatigue or pain [1,2]. After the 2003 severe acute respiratory syndrome (SARS) outbreak, about one-third of the infected population developed reduced tolerance to exercise, despite having normal lung function, which is similar to what has been observed in post–COVID-19 syndrome [3,4]. However, the first SARS outbreak only affected 8000 people locally while COVID-19 has affected more than 750 million people worldwide according to the World Health Organization (WHO) [5]. With a prevalence of 54% for hospitalized and 34% for nonhospitalized patients, post–COVID-19 syndrome is of major concern for public health [6].

Post–COVID-19 syndrome or long COVID is a condition where patients experience persisting general unspecific symptoms like fatigue, myalgia, and concentration or sleep disturbance even several months after a SARS-CoV-2 infection [4]. While the mechanisms underlying the persisting symptoms are not all well understood, it has been shown that the neurotropism of SARS-CoV-2 can lead to neural damage and persisting neurologic symptoms including neuropathic pain, transient memory loss, and olfactory dysfunction [7-9]. Very similar to previously described postviral conditions like myalgic encephalomyelitis or chronic fatigue syndrome, the post–COVID-19 symptoms often fulfill classification criteria for fibromyalgia [10,11]. Moreover, recent studies suggest that fibromyalgia could be triggered by COVID-19, thus becoming a postviral chronic condition [12,13].

### Digital Health as a Therapeutic Approach

While these chronic syndromes affecting a significant part of the population are known to be difficult to treat with traditional medication, digital health solutions could have great potential. Digital health solutions have recently proven efficacy in chronic pain and fibromyalgia management [14,15]. Self-management plays a big role in the empowerment of these patients, and encouraging studies showed the efficacy of such programs [16,17]. Moreover, cognitive behavioral therapy or mindfulness and education training have shown substantial positive effects in a self-management setting, thus empowering the patients by involving them in the therapeutic process [18-20]. Based on these observations and findings, the development of a self-management platform that includes similar therapeutic approaches for patients with post–COVID-19 syndrome could be valuable.

### The Challenge of Adoption

Treatment adherence is a major issue in chronic disease management. Since digital health solutions require greater effort (time and dedication) from patients in comparison to traditional medication, efforts have to be made to ensure high adoption. However, very few efforts are made to improve patient engagement in digital health, where the focus is usually set on the assessment of the therapeutic value through clinical studies. This lack of consideration for adoption and engagement is certainly responsible for the limited adoption of digital health solutions by patients and physicians, despite the plethora of apps available and the increasing compliance of health care systems for these solutions. Difficulties in distinguishing validated therapies from lifestyle solutions play a role in this phenomenon as well [21]. While studies have shown that barriers to adherence were mostly fear of adverse effects, misperception of therapeutic value, forgetfulness, costs, and poor patient-physician interaction in conditions like rheumatoid arthritis, the role of patient preferences emerges as one of the pivotal factors for adherence [22,23].

### Patient Centricity in the Development Process

In recent years, the health care industry has increasingly recognized the importance of patient centricity in the development of health care solutions [24]. The successful adoption and use of digital health solutions, particularly for complex and chronic conditions such as fibromyalgia, heavily rely on incorporating patient perspectives and preferences throughout the development process. To address the challenges of adoption and enhance the usability and effectiveness of digital health solutions, it is crucial to engage patients as active participants in the design and evaluation stages. User experience–based approaches, such as usability testing and patient preference surveys, have emerged as valuable tools to gather insights and guide the development of patient-centered digital health solutions [25]. By involving patients in the research process, we can gain a deeper understanding of their needs, preferences, and expectations, ensuring that the resulting product is tailored to meet their unique needs. Therefore, in this study, we aim to explore the integration of patient preference techniques in the development of a digital health solution for patients enduring from fibromyalgia-like symptoms after a viral infection, leveraging user experience research to enhance usability and patient satisfaction.

### Regulatory Requirements and Usability

On the regulatory side, requirements play a crucial role in the development of digital health products, particularly when it comes to ensuring usability and facilitating widespread adoption. Formative evaluations and usability testing are essential components of the development process, enabling the identification and mitigation of usability issues early on. For instance, the International Organization for Standardization (ISO) 13485 standard provides guidelines for quality management systems in the medical device industry, emphasizing the importance of usability engineering and human factors [26]. Additionally, the certification process for software as a medical device involves demonstrating compliance with regulatory requirements, including usability considerations. By adhering to these norms and engaging in rigorous formative evaluations and usability testing, developers can not only ensure compliance but also enhance the overall user experience, thereby increasing the likelihood of adherence and adoption of digital health products.

### Objectives

This study aims to leverage patient preferences as a guiding framework for the development of a digital health self-management platform tailored specifically for patients with postviral fibromyalgia-like symptoms. By using patient preference surveys and conducting usability testing, we seek to identify the unique needs, preferences, and challenges faced by this patient population. Through a comprehensive understanding of these factors, we aim to develop an app that aligns with patient expectations, fosters engagement, and enhances self-management practices. This research will contribute to the growing field of patient-centered digital health interventions and provide valuable insights for health care professionals, developers, and stakeholders involved in the development and implementation of digital health solutions for chronic conditions. Ultimately, our goal is to improve the adherence and adoption rates of digital health interventions, leading to better patient outcomes and enhanced quality of life for the patients.

## Methods

### Study Design

This is an exploratory study that aims to actively involve patients in the development process of a digital health app named “POCOS” through patient preference surveys and usability testing. This design has been chosen to gain a comprehensive understanding of the unique needs, preferences, and challenges faced by the target population.

### Participants

Recruitment for the patient preference surveys was conducted through a collaboration with the Swiss patient post–COVID-19 association, Long COVID Schweiz [27]. The association’s coordinator conducted a preliminary screening to identify members who met the study criteria—those who had experienced a COVID-19 infection and were currently experiencing persisting fibromyalgia-like symptoms. Interested individuals were then contacted through email, resulting in 53 and 33 participants responding to the first and second surveys, respectively.

For the usability testing, potential participants were identified from the cohort of patients engaged in the multimodal care program at Lausanne University Hospital’s Rheumatology Department, which caters to patients with fibromyalgia. Eligibility requires patients to exhibit fibromyalgia-like symptoms persisting after a viral infection, predominantly COVID-19. Selected patients were approached for participation during their care program, and those who consented underwent usability testing. This process was conducted in a face-to-face setting by the study nurse. The sample consisted of 6 patients (4 women and 2 men, aged between 20 and 59 years) who fulfilled the inclusion criteria and participated in the usability testing.

### Data Collection

Survey data were collected using web-based questionnaires administered through the SurveyMonkey (SurveyMonkey Inc) platform. The survey items were developed based on a comprehensive review of existing literature, expert input, and previous studies in the field.

In total, 2 surveys were sent to patients, the first (survey 1, fifty-three respondents) focused on user preferences, design aspects, and symptom reporting, and the second (survey 2, thirty-three respondents) focused on patient expectations regarding content or usability.

Usability testing data have been collected by a trained nurse during interviews with the participants. These data consist of notes taken from observation of the patients using the newly developed primary prototype of the app by the nurse and reported feedback that patients have given to the nurse during and after the use of the app.

### Ethical Considerations

This study adhered strictly to ethical standards for human participant research. Ethical review and approval were obtained from the commission cantonale d’éthique de la recherche sur l’être humain (2021-01680), and all participants provided permission for the use of their data in secondary analyses without the need for additional consent. We ensured the privacy and confidentiality of all study participants by anonymizing or deidentifying all personal data before analysis. Participants did not receive any compensation for their involvement in the study. Furthermore, to protect participant identity, no personal identifiers are present in any images within the study or Multimedia Appendix 1.

## Results

### Patient Preference Survey (Survey 1)

**Figure 1:**
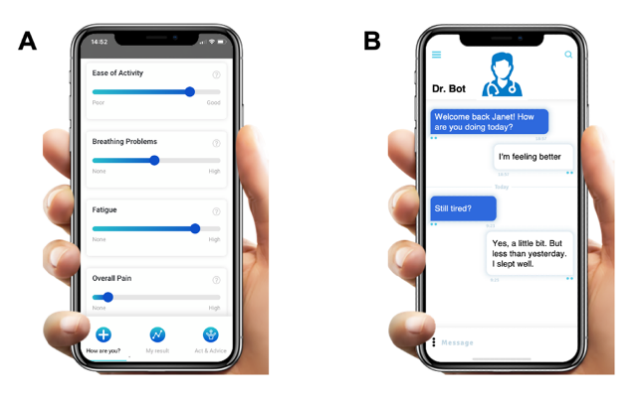
Patient-reported outcome designs. (A) Symptoms are listed with sliders and intensity scales from 1 to 10. (B) The automated chatbot regularly asks the user for symptoms entry through text (survey 1, number of respondents is 53).

Participants were asked about the patient-reported outcome design they would prefer between a symptoms list with intensity scales from 1 to 10 or an automated chatbot asking for symptoms entry (Figure 1). In total, 48 out of 53 (91%) respondents found the symptoms list more appropriate according to their conditions.

Patients were then asked about the number of symptoms- and activity-reporting questions they would like to answer regularly. For both types of questions, most of the respondents preferred 5-10 questions (n=37, 70% and n=31, 58%, respectively). A total of 15 (28%) and 10 (19%), respectively, would like to answer more than 10 questions while 1 (2%) and 12 (23%) preferred to answer 5 questions or less (number of respondents is 53).

The most relevant symptoms patients would like to regularly report considering their conditions are respectively, among the list, fatigue, concentration level, sleep quality, memory level, pain, and more specifically pain location (Table 1).

Among the most relevant activities they would like to report according to their daily life with post–COVID-19 are physical activities, nonmedical therapies like yoga, massage or relaxation, doctor visits, and painkiller use (Table 2).

**Table 1:** Most relevant symptoms patients would like to regularly report on the platform considering their post–COVID-19 syndrome conditions (survey 1, number of respondents are 53).

Symptoms patients would like to regularly report on the platform	Frequency (N=53), n (%)
Fatigue level	48 (91)
Concentration level	36 (68)
Sleep quality	33 (62)
Memory level	30 (57)
Pain location	28 (53)
Ease of activity	26 (49)
Breathing problems	21 (40)
Specific pain level	20 (38)
General health level	18 (34)
Mood	16 (30)
Gastrointestinal problems	16 (30)
Anxiety level	15 (28)
Overall pain level	14 (26)
Smelling and taste problems	9 (17)
Others: tinnitus, brain fog, postural orthostatic tachycardia syndrome, nausea, dizziness, and menstrual problems	15 (28)

**Table 2:** Most relevant activities patients would like to regularly report on the platform according to their daily life with post–COVID-19 syndrome (survey 1, number of respondents are 53).

Most relevant activities patients would like to report on the platform	Frequency (N=53), n (%)
Physical activity	48 (91)
Nonmedical therapies	42 (79)
Doctor visits	28 (53)
Painkiller use	24 (45)
Nutrition	19 (36)
Others: social activities, housekeeping, job activity, psychological visits, and relaxation	15 (28)

### Patient Expectations Survey (Survey 2)

Questions about what patients would like to monitor on the platform and what kind of therapeutic content they would expect from it have been asked. While there was a disparity in what patients would like to visualize on the platform, the evolution of their symptoms and energy level were the most frequently chosen answers. In terms of therapeutic content, most of the patients expect to be informed about what has helped people with similar symptoms, to be provided with a therapeutic exercise program, with links to patient communities, and with information and news about their conditions (Table 3).

Finally, more technical questions like their expected frequency of use, time spent on the platform, and on which support they would use it, were asked. Answers showed that most of the patients would use the platform every day, from 5 to 10 minutes on their mobile phones (Table 4).

**Table 3:** Response frequency to questions by patients with post–COVID-19 syndrome (survey 2).

Responses to the questions		Frequency, n (%)
**What would the patients like to visualize on the platform (n=26)**		
	In total, 3 main symptoms and evolution	7 (27)
	Every symptom and evolution	14 (54)
	List of symptoms that get better or worsen	15 (58)
	Energy level evolution	19 (73)
	Disease activity evolution	12 (46)
	Average disease activity score of other users	4 (15)
	Their activity history	11 (42)
	Physical activity evolution	13 (50)
	Body mass evolution	1 (4)
	Others: activities versus symptoms or system versus symptoms intensity charts	2 (8)
**What type of therapeutic content patients expect from the platform (n=27)**		
	Personalized therapeutic exercises	16 (59)
	Informative content about symptoms and the disease	15 (56)
	What has helped people with similar symptoms	22 (82)
	Training program	16 (59)
	Advice from experts in the field	15 (56)
	Links to communities of patients or patients’ centers	17 (63)
	Information, news, and articles about the disease	16 (59)
	Quizzes and tools	4 (15)
	Discussion forum with the “POCOS” community	13 (48)
	Others	0 (0)

**Table 4:** Frequency at which patients expect to use the platform, time patients expect to spend on the platform per use and support patients expect to use the platform (survey 2, the number of respondents is 33, 33, and 32, respectively).

		Frequency, n (%)
**Frequency patients expect to use the platform (n=33)**		
	Less than once a week	1 (3)
	Once a week	6 (18)
	3 times a week	3 (9)
	5 times a week	3 (9)
	Everyday	19 (58)
	Several times a day	1 (3)
**Time patients expect to spend on the platform per use (n=33)**		
	Less than 5 minutes	4 (12)
	5 to 10 minutes	19 (58)
	10 to 20 minutes	6 (18)
	20 to 30 minutes	4 (12)
	30 minutes to 1 hour	0 (0)
	More than 1 hour	0 (0)
**Support patients expect to use the platform on (n=32)**		
	Mobile phone	27 (84)
	Tablet	2 (6)
	Computer	3 (9)
	Television	0 (0)
	Others	0 (0)

### Usability Testing

Out of the 6 participants, only 2 showed a sufficient understanding of the functionalities and managed to navigate through the app without additional help. In total, 2 patients did not even manage to register and start to use the app due to lack of guidance and information, and the 2 other patients showed some difficulties in understanding the workflow and efforts required to navigate. Only 1 patient was satisfied with the app and the content, reported it to be intuitive, easy to use, and recommended it to peers. Several patients reported a lack of comprehension of the app features and found that some of the therapeutic content was not specific enough.

## Discussion

The patient preference survey results are insightful regarding patients’ needs for symptom and physical activity reporting. Clear preferences emerged for a list-based approach over an automated chatbot for symptom documentation. A majority opted to respond to 5-10 questions, suggesting a moderate yet flexible reporting frequency could be most beneficial. This preference for moderation extends to activity reporting, supporting the need for adaptable digital health tools (Figure 1).

The survey findings emphasize the significance of tracking key health symptoms—fatigue, concentration, sleep quality, memory level, and pain location—which are reported by over half of the participants (as detailed in Table 1). These symptoms align with documented post–COVID-19 syndrome effects in existing literature [4,28,29]. In addition to symptom monitoring, the survey underlines the critical role of physical activity reporting in the context of fibromyalgia-like symptoms management. A substantial majority, exceeding 90%, expressed a desire to consistently log their physical activity, indicative of a widespread recognition of its contribution to overall health and well-being. This observation is supported by research demonstrating the beneficial impacts of physical activity on improved physical function, reduced risk of chronic diseases, and enhanced mental health [30-32]. Regular assessment of physical activity, therefore, could yield insights into patient health, guiding more informed management of postviral fibromyalgia-like symptoms.

Most patients indicated a strong desire to track the progression of their symptoms and energy levels visually. They also expected the platform to offer diverse therapeutic content, with a significant portion wanting information on effective strategies that have aided others with similar symptoms (82%). Additionally, they anticipated the inclusion of community support links, as well as exercise and educational programs (Table 3).

Usability testing identified significant onboarding challenges and revealed gaps in patients’ understanding of the app’s functions. Further research should be performed on the onboarding process to investigate its impact on usability, adoption, and retention rate by implementing and testing different onboarding strategies.

This study’s insights should be interpreted in the context of certain limitations. The participant pool was drawn exclusively from a Swiss patient post–COVID-19 association, which may not reflect the broader demographic affected by postviral conditions. The modest sample size, especially for usability testing, may limit the extrapolation of our findings to larger populations. Further research with a more diverse and extensive sample would be beneficial to validate and expand on our results.

In conclusion, the integration of patient preferences into the design of digital health apps has been substantiated by the findings of this study. The data derived from patient surveys have been instrumental in shaping the development of an app for managing postviral fibromyalgia-like symptoms (Figure S1 in Multimedia Appendix 1). These insights not only facilitated a tailored app that aligns with user expectations but also highlighted the significance of patient engagement and the potential for improved health outcomes.

The advancement of patient-centered digital health solutions, as evidenced by the app developed in this study, underscores a paradigm shift toward more responsive and adaptive health technology design. This approach is anticipated to enhance patient adherence and the efficacy of digital health interventions. The broader implications of this research advocate for the incorporation of user experience research early in the development cycle, ensuring that digital health technologies are congruent with the needs and preferences of end users.

## Appendix

Multimedia Appendix 1 POCOS user interface (app). Left side: "How are you?" with electronic patient-reported outcomes and activity. Middle: "My result" with monitoring of symptom activity and health conditions. Right side: "Act and Advice" with personalized training program, adapted to the user’s symptoms.

## Data Availability

All data generated or analyzed during this study are included in this published article (and in Multimedia Appendix 1).

## Abbreviations

ISO: International Organization for Standardization
SARS: severe acute respiratory syndrome
WHO: World Health Organization
