# Supplementary figures and images for: Development of a Management App for Postviral Fibromyalgia-Like Symptoms: Patient Preference-Guided Approach
Source: JMIR Form Res. 2024 Apr 19;8:e50832. doi: 10.2196/50832 (PMC11069091; doi:10.2196/50832)

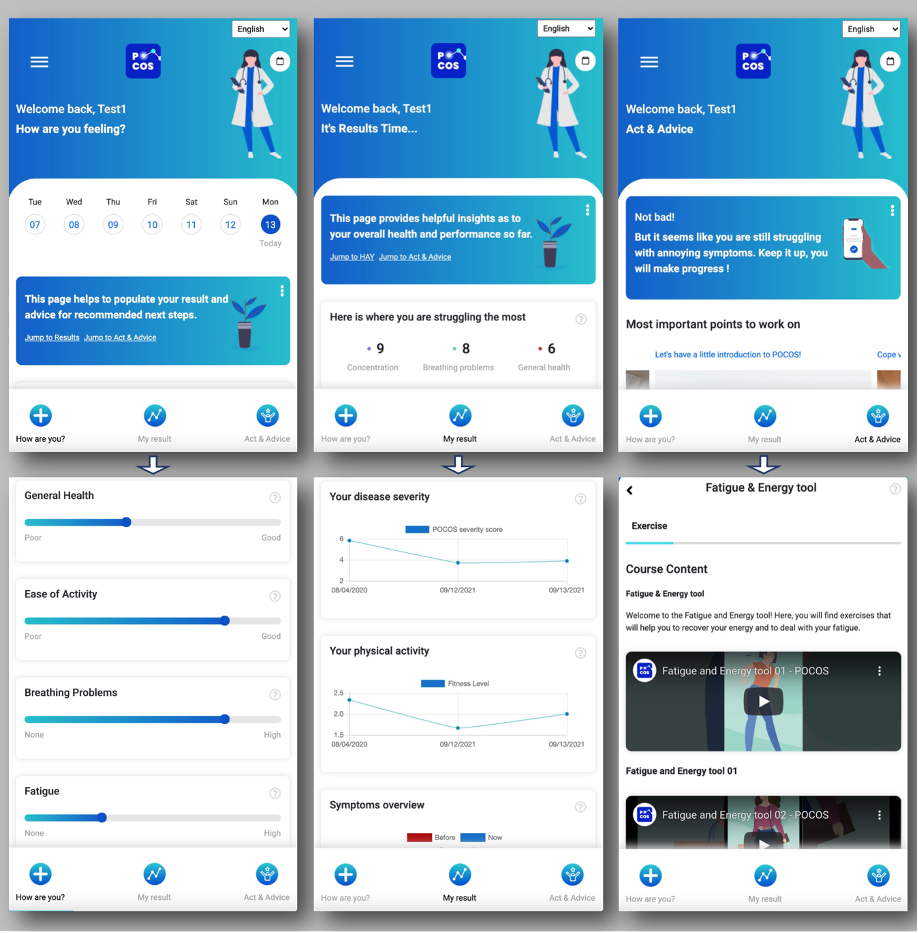

Supplement: Multimedia Appendix 1 [file formative_v8i1e50832_app1.png]
